# A novel spectral entropy-based index for assessing the depth of anaesthesia

**DOI:** 10.1186/s40708-021-00130-8

**Published:** 2021-05-12

**Authors:** Jee Sook Ra, Tianning Li, Yan Li

**Affiliations:** School of Sciences, University of Southern Queensland, West St, Darling Heights, Toowomba, QLD 4350 Australia

**Keywords:** Spectral entropy, EEG, Depth of anaesthesia, Machine learning, Linear regression

## Abstract

Anaesthesia is a state of temporary controlled loss of awareness induced for medical operations. An accurate assessment of the depth of anaesthesia (DoA) helps anesthesiologists to avoid awareness during surgery and keep the recovery period short. However, the existing DoA algorithms have limitations, such as not robust enough for different patients and having time delay in assessment. In this study, to develop a reliable DoA measurement method, pre-denoised electroencephalograph (EEG) signals are divided into ten frequency bands (*α, β1, β2, β3, β4, β, βγ, γ, δ* and *θ*), and the features are extracted from different frequency bands using spectral entropy (SE) methods. SE from the beta-gamma frequency band (21.5–38.5 Hz) and SE from the beta frequency band show the highest correlation (R-squared value: 0.8458 and 0.7312, respectively) with the most popular DoA index, bispectral index (BIS). In this research, a new DoA index is developed based on these two SE features for monitoring the DoA. The highest Pearson correlation coefficient by comparing the BIS index for testing data is 0.918, and the average is 0.80. In addition, the proposed index shows an earlier reaction than the BIS index when the patient goes from deep anaesthesia to moderate anaesthesia, which means it is more suitable for the real-time DoA assessment. In the case of poor signal quality (SQ), while the BIS index exhibits inflexibility with cases of poor SQ, the new proposed index shows reliable assessment results that reflect the clinical observations.

## Introduction

Monitoring the patient’s depth of anaesthesia (DoA) is one of the current challenges in medicine. An accurate assessment of the DoA is crucial as a patient under-dosage may lead to intraoperative awareness with recall, while over-dosage may lead to prolonged recovery and an increased risk of postoperative complications. Various human and animal researchers confirmed that electrical brain activities significantly correlated with the DoA during surgery. Most brain electrical activities can be represented by the electroencephalograph (EEG) signals. EEG monitoring methods are typically non-invasive, with small metal discs with thin wires (electrodes) placed on the scalp. Then, signals (voltage fluctuations resulting from ionic current within the brain's neurons) are sent to a computer to record the results. EEG patterns change during stages of anaesthesia, and as the level of anaesthesia becomes deeper, EEG signals gradually shift towards higher-amplitude and lower-frequency activity. The DoA monitoring using EEG improves the outcomes of the patient treatment by reducing the incidences of intraoperative awareness, minimizing anaesthetic drug consumption and resulting in faster wake-up and recovery [1, 2]. Consequently, most of the recent research has focused on developing and finding non-invasive ways to monitor the DoA based on brain electrical activities.

When using EEG signals to measure the DoA, the bispectral index (BIS) monitor is commonly the primary indicator used by anesthesiologists. The BIS index is a statistically based, empirically derived complex parameter, which is a weighted sum of several EEG sub-parameters, including a time domain, frequency domain, and high order spectral sub-parameters [3]. The BIS takes an EEG complex signal and provides the result into a single dimensionless number (index), ranging from 0 (almost flat EEG activity) to 100 (awake). An appropriate depth level for general anaesthesia occurs when the BIS index is between 40 and 60 [4].

However, the BIS has limitations, such as being delayed, not working efficiently with different anaesthesia medications, and not being accurate across patients [1]. BIS and other existing monitors showed a long-time delay after a change in a state of consciousness [5]. There are some possible improvements in the algorithms. Various methods have been developed to decompose and extract features of a frequency segment of the raw EEGs over the recent years. One is to measure an entropy of a signal, which is a powerful means to quantify the randomness in a dataset [6]. Various entropy algorithms have been used in clinical studies, but until now, the entropy measurement has rarely been applied to different frequency bands of anaesthetic EEG signals. Thus, it is not clear which entropy is sensitive to which frequency bands in anaesthetic EEG signals. The entropy application to the specific frequency bands can improve the DoA assessment as it can reduce the interference from other frequency bands. This research applies spectral entropy (SE) from the decomposed EEG signals to extract features from different frequency bands of EEG signals for an effective DoA assessment. A window segmentation technique [7] is employed with decomposing frequency bands of an EEG signal, and then each EEG segment is divided into a number of small blocks. The parameters for SE are calculated from the blocks and averaged over each segment. Then, the selected parameters are trained, tested, and evaluated by the Pearson correlation coefficient to build a new DoA index model. Our findings show that the SE of the beta-gamma frequency band (21.5–38.5 Hz) and SE of the beta frequency band provide the highest R-squared value of 0.8458, in consistent with the BIS values.

The remainder of the paper is arranged as follows. Section 2 presents a brief literature review about the proposed techniques for the DoA measurement. Section 3 explains the datasets used in this paper, experimental setup and results. Section 4 discusses the findings of this research. Finally, the conclusions of this study are drawn in Sect. 5.

## Methods

**Fig. 1:**
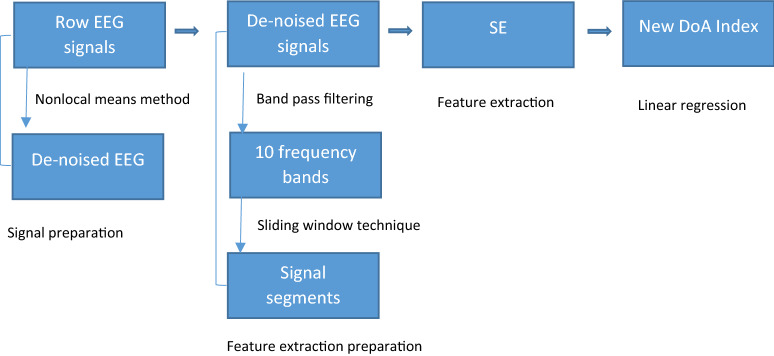
The diagram of the new DoA index design and evaluation based on EEG signals

The original EEG signals used in this research were denoised using a nonlocal means method [2]. EEG signals are hard to process due tso their high complexity and non-stationarity. Decomposing an EEG signal into a set of subsets with different frequency bands is an efficient strategy to analyse it. Firstly, an EEG signal is partitioned into small segments using a window segmentation technique [7]. The window size in this paper was 56 s (s) with overlapping of 55 s. EEG segment was divided into a number of blocks. The SE parameters are calculated from the above blocks and averaged over each segment. These values can be used in time-domain methods to calculate their correlations with their changing anaesthetic states. In this paper, the method for a new DoA index design is depicted in Fig. 1.

### EEG data processing

In this study, the anaesthetic EEG signals are decomposed into sub-frequency bands through a fast Fourier transform (FFT) which is an efficient band filtering method with low computational intensity. The parameter dynamics are based on the decomposed frequency bands in this research. Research shows that EEG signals collected from the scalp can reflect patients' anaesthetic states. Various methods have been used to extract useful segments for the raw EEG analysis in recent literature. Bayesian learning from frequency bands [8, 9] is proposed to simultaneously optimize spectral filters and spatial filters along with a modified factored-sampling method. Wavelet transformation (WT) is one of the popular segment decomposing methods, which usually includes an orthonormal WT and an integral wavelet [10, 11]. WT enables segment detection in both time and frequency responses of finite duration signal components. FFT is also one of the popular analysis methods for processing EEG data. FFT decomposes linear differential equations with non-sinusoidal source terms and breaks them down into component equations (with sinusoidal source terms) that transform data into frequency domain variables. For example, the human emotion recognition study by Murugappan and Murugappan [12] framed EEG signals into a short time duration of 5 s, and two statistical features (spectral centroid and SE) in four frequency bands, namely alpha (8 Hz–16 Hz), beta (16 Hz–32 Hz), gamma (32 Hz–60 Hz) and alpha to gamma (8 Hz–60 Hz) are extracted using FFT [12]. Applying a simple classifier such as *K-*nearest neighbour (KNN) with that frequency domain offered a maximum mean classification accuracy of 91.33% in the beta band [12]. The benefit of the FFT algorithm is that the computational time is reduced [13], which makes the FFT applicable in a real-time DoA measurement.

### Spectral entropy

This research applies spectral entropy (SE) of the decomposed EEG signals to extract features from frequency bands of EEG signals for the effective DoA assessment. Extracting representative features simplifies the amount of data needed to describe a huge set of data. Feature extraction is also important to minimize the loss of essential information embedded in a signal. Various methods have been used to extract features from EEG signals. Popular methods are entropy [14], detrended moving average (DMA) [15], isomap-based estimation [16], Bayesian [17], and others [18]. In the past decade, entropy algorithms have been widely used for features extraction in anaesthetic EEG signals. EEG patterns during the course of anaesthesia are time series and nonlinear. An entropy algorithm is a measure of complexity that can be applied to any types of time series and nonlinear data, including physiological data, such as heart rate variability and EEG data. One of the entropy methods, SE, quantifies the amount of potential information conveyed in the power spectrum of a given signal. Zhang et al. [19] evaluated the inter-session prediction performance of a sensorimotor rhythm-based brain–computer interface using a SE predictor. Their results showed that the average classification accuracy of the inter-session prediction is up to 89% [19]. Das and Bhuiyan also investigated the efficiency of several SE-based features in a comprehensive analysis of focal and non-focal EEGs [20]. When the log-energy entropy values were utilized as features in a KNN classifier to classify their EEG signals, it provided an 89.4% accuracy and 90.7% sensitivity, which were higher than those by some state-of-the-art methods [20]. Xu et al. [21] studied the SE from rats' EEG to investigate and measure brain activity variations under different depths of anaesthesia. They found that the SE of EEG would decrease quickly while the DoA was from light to deep and vice versa [21]. However, despite numerous researches engaged with entropy-based algorithms, few articles were reported to use SE for human-related DoA assessment. Hence, this research examines the SE of each frequency band from an EEG signal and also investigates a PE to compare their performances from features extraction.

The SE of a signal is a measure of its power spectrum distribution [22]. The SE takes the signal's normalized power spectrum distribution in the frequency domain as a probability distribution and calculates its Shannon entropy. The equations for SE are derived from the equations for the power spectrum and probability distribution for a signal *x(n),* where *n* is a sequence of number. For a signal *x(n),* the power spectrum is *S(m)* =*|X(m)|*^*2*^, where *X(m)* is the discrete Fourier transform of *x(n)* and *m* = 0, 1, 2, *… n*−1. According to Ulrych [22], the probability distribution *P(m)* is then:

1 $$P( m ) = \frac{S( m )}{{\sum\nolimits_{i} {S( i )} }}.$$

The SE (*H)* follows as:

2 $$H = - \sum\limits_{m = 1}^{N} {P\left( m \right)\log_{2} P\left( m \right)} .$$

Normalizing

3 $$H_{n} = - \frac{{\sum\limits_{m = 1}^{N} {P\left( m \right)\log_{2} P\left( m \right)} }}{{\log_{2} N}},$$

where *N* is the total frequency points. The denominator, *log*_*2*_*N*, represents the maximal SE of the white noise, uniformly distributed in the frequency domain. If a time–frequency power spectrogram *S*(*t*, *f*) is known, then the probability distribution *P(m)* becomes:

4 $$P\left( m \right) = \frac{{\sum\nolimits_{t} {S\left( {t,m} \right)} }}{{\sum\nolimits_{f} {\sum\nolimits_{t} {S\left( {t,f} \right)} } }},$$

where *t* is time, and *f* is frequency. Then the SE at time *t* is:

5 $$H\left( t \right) = - \sum\limits_{m = 1}^{N} {P\left( {t,m} \right)} \log_{2} p\left( {t,m} \right).$$

### Features extraction based on SE

The SE is calculated from blocks in a segmented EEG and averaged over each segment. The SE values in time domain analysis methods should be highly correlated with changing anaesthetic states. These correlations should also be robust for different patients. The degree of their correlation is measured by the coefficient of determination (*R*^*2*^) in this research. *R*^*2*^ indicates the degree of the variance in a dependent variable. The SE values are calculated from 10 frequency bands (*α, β1, β2, β3, β4, β, βγ, γ, δ,* and *θ*) of each EEG episode, and *R*^*2*^ is used to evaluate the correlation between parameters and anaesthetic states (referring to the BIS in this study). The definition of *R*^*2*^ [23] is:

6 $$R^{2} = 1 - \frac{{\sum\nolimits_{i} {\left( {y_{i} - f_{i} } \right)^{2} } }}{{\sum\nolimits_{i} {\left( {y_{i} - \overline{y}} \right)^{2} } }},$$

where *y*_*i*_ is a data set, $\overline{y }$ is the mean of a data set, and *f*_*i*_ is a set of predicted values. The greater the *R*^*2*^ value is, the higher the correlation between the parameter and the BIS value is.

### Regression models and evaluation

Machine learning algorithms have been widely used in signal classification. Four different machine learning algorithms of a linear regression, a support vector machine (SVM), a rectifier-based deep learning and a neural network are applied for performance comparisons using the training datasets, and are evaluated using the testing datasets. These four machine learning methods are popular and commonly used to analyse EEG data. The calculated parameters from features extraction are utilized for training a model to find out how  a single feature or different combinations of features can discriminate between distinct stages of anaesthesia. A combination of features from EEG waveforms in time-domain or band powers in the frequency domain can describe the difference among anaesthetic states. To characterize these states, a set of optimum EEG features are extracted using frequency discrimination methods, and these features establish a relationship between input and output variables, that may be suitable for various linear or nonlinear analysis. For example, Yildirim et al. [24] combined four fundamental ensemble learning methods of bagging, boosting, stacking, and voting with five different machine learning algorithms of a neural network, an SVM, a KNN, Naive Bayes, and C4.5 with the most optimal features extracted from EEG signal data sets for the DoA assessment [24]. Some research employed a single model and still achieved high accuracy. Das and Bhuiyan [20] utilized log-energy entropies as features with a KNN classifier to classify EEG signals. It provided 89.4% accuracy with 90.7% sensitivity [20]. Liang et al. [25] used a genetic algorithm and an SVM to identify the emergence of EEG patterns. The accuracy obtained by the GA-SVM was between 90.64 and 72.86% [25]. Linear regression has also been widely used for the DoA assessment [26, 27].

Regression analysis consists of a set of machine learning methods that allow us to predict a continuous outcome variable (*y*) based on the value of one or multiple predictor variables (*x*). Once features are extracted, a regression technique is employed to evaluate the correlation between the predicted outcome by the model and the changing of the anaesthetic states, which is referred to the BIS value. The Pearson correlation coefficient (*r*) and the root mean squared error (*RMSE*) are used to evaluate the correlation between the new index and the BIS index. The definition of *r* [28] is given below:

7 $$r = \frac{{\sum {\left( {x_{i} - \overline{x}} \right)\left( {y_{i} - \overline{y}} \right)} }}{{\sqrt {\sum {\left( {x_{i} - \overline{x}} \right)^{2} \left( {y_{i} - \overline{y}} \right)^{2} } } }},$$

where *x* is the new index value, $\overline{x }$ is the mean of the new index, *y* is the corresponding BIS value, and $\overline{y }$ is the mean of the BIS index. The value of *r* is between [−1 1]. If *r* is closed to 1 or −1, it means that the two indexes are highly correlated. If *r* equals 0, it means that there is no correlation at all between the indexes.

The *RMSE* is a square root of *MSE* [29]. The definition of the *MSE* is as follows:

8 $${\text{MSE = }}\frac{1}{n}\sum\limits_{i = 1}^{n} {\left( {Y_{i} - \hat{Y}_{i} } \right)}^{2} ,$$

where *n* is the number of the data points, ${Y}_{i}$ is a set of the observed values, and ${\widehat{Y}}_{i}$ is a set of the predicted values.

In this research, a scatter plot is employed to identify the correlational relationship between the BIS and the values of SE calculated from EEG signals before establishing the new DoA index. A scatter plot uses dots to represent two numeric variables. Its primary uses are to observe and show relationships between the values of two variables.

## Results

### Experimental data and EEG data preprocessing

The EEG data were collected at Toowoomba St Vincent’s Hospital from 24 adult patients. The demographic information of all the participants involved in this study is explained in Table 1. Their typical drug administration included earlier pharmaceuticals, intravenous midazolam 0.05 mg/kg, fentanyl 1.5–3 μg/kg or alfentanil 15–30 μg/kg.

**Table 1:** Patient demographics and intraoperative drug usage

Age (year)	Weight (kg)	Height (cm)	Gender (F/M)	Midazolam (mg)	Alfentanil (μg)	Propofol (mg)	Parecoxib (mg)	Fentanyl (μg)
2–83	55–130	154–194	15/22	2–5	500, 750, 1000	90–200	40	100, 150

The EEG signals are typically classified into five basic frequency bands that are *α* (8 to 16 Hz), *β* (16 to 32 Hz), *γ* (32 to 60 Hz), *δ* (1 to 4 Hz) and *θ* (4 to 8 Hz) [5]. In this research, the EEG characteristics analysis is based on the different frequency bands, and the DoA algorithm is designed upon the frequency bands dynamics. For the BIS index, the phase coupling between high frequency (40 to 47 Hz) and a broader frequency range (0.5 to 47 Hz) of EEG waves is quantified, and the ratio of higher frequency waves (30 to 47 Hz) to other waves of lower frequency (11 to 20 Hz) is measured to compute the bispectrum [30]. To further explore the relationship between the SE values and frequency bands, the EEG signals are divided into five basic frequency bands (*α, β, γ, δ* and *θ*) and four small *β* bands and *βγ* (21.5 to 38.5 Hz) by the FFT. Small *β* bands are *β1* (13 to 17 Hz)*, β2* (17 to 21.5 Hz), *β3* (21.5 to 26 Hz), and *β4* (26 to 30 Hz) (Fig. 4)*.*

**Fig. 2:**
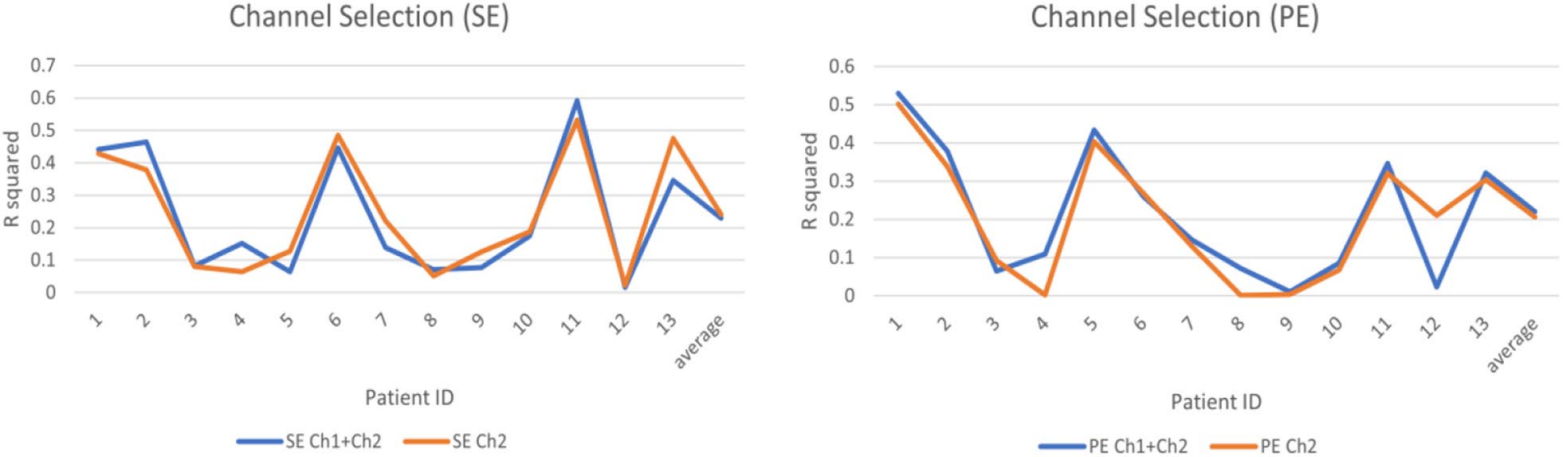
The *R*^*2*^ value of SE and PE from Ch1 + Ch2 EEG and Ch2 EEG (the reference is the BIS index)

**Fig. 3:**
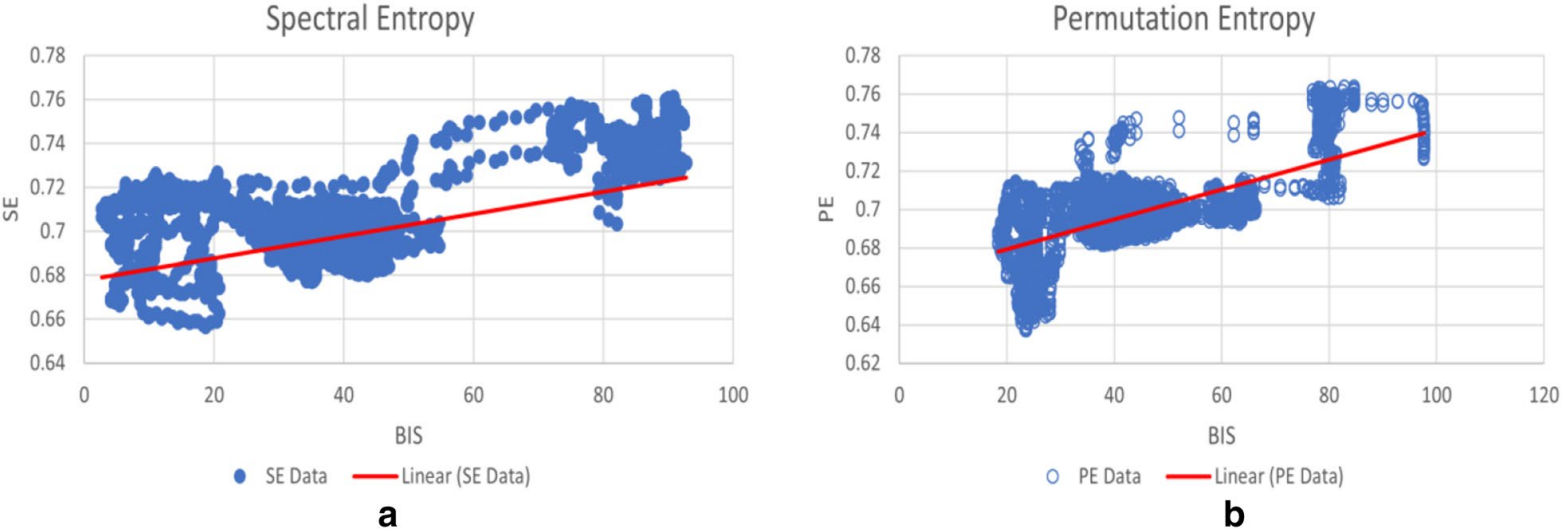
The relationships of SE and PE with the BIS value: **a** SE, **b** PE. The best-fit line is red. The fitted linear relation indicates that the two methods are correlated (for Patient ID 10)

**Fig. 4:**
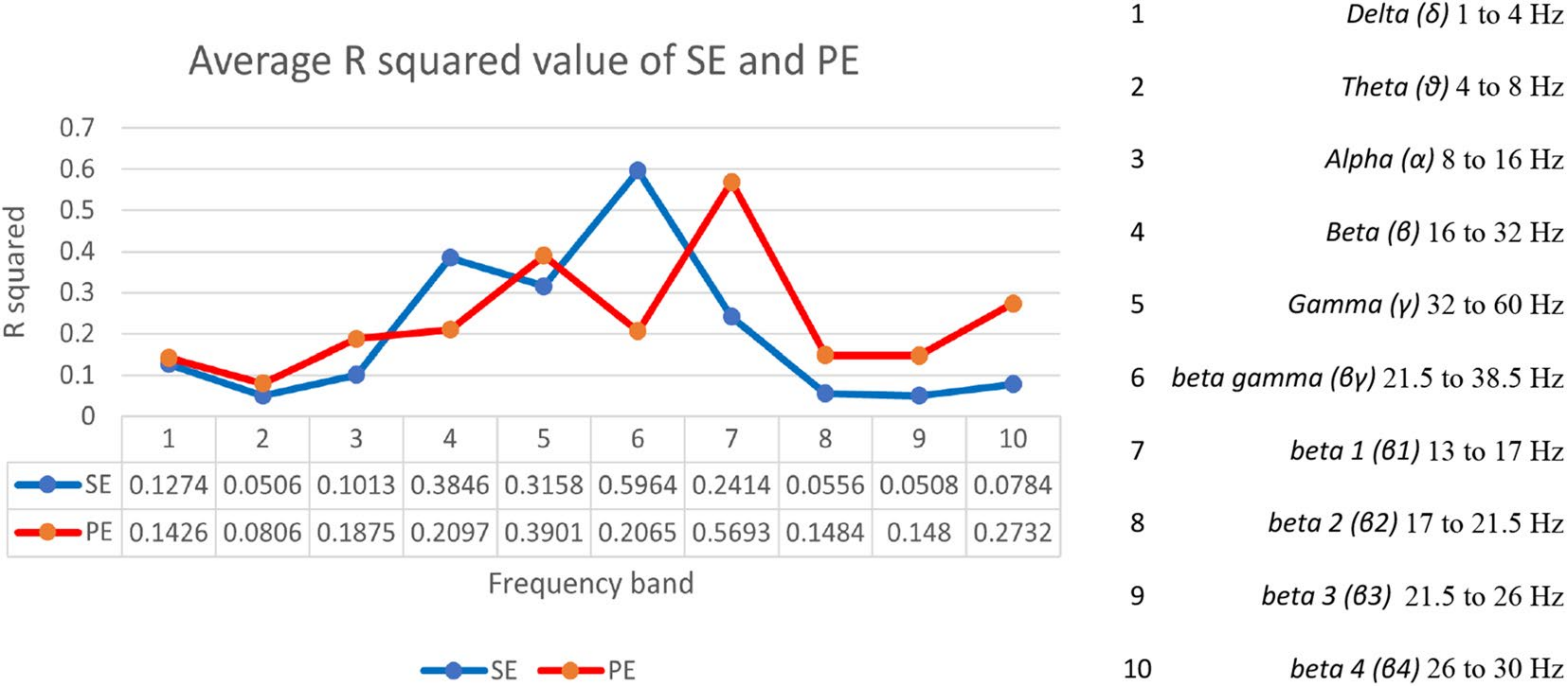
Comparison between SE and PE from different frequency bands (the reference is the BIS index)

**Fig. 5:**
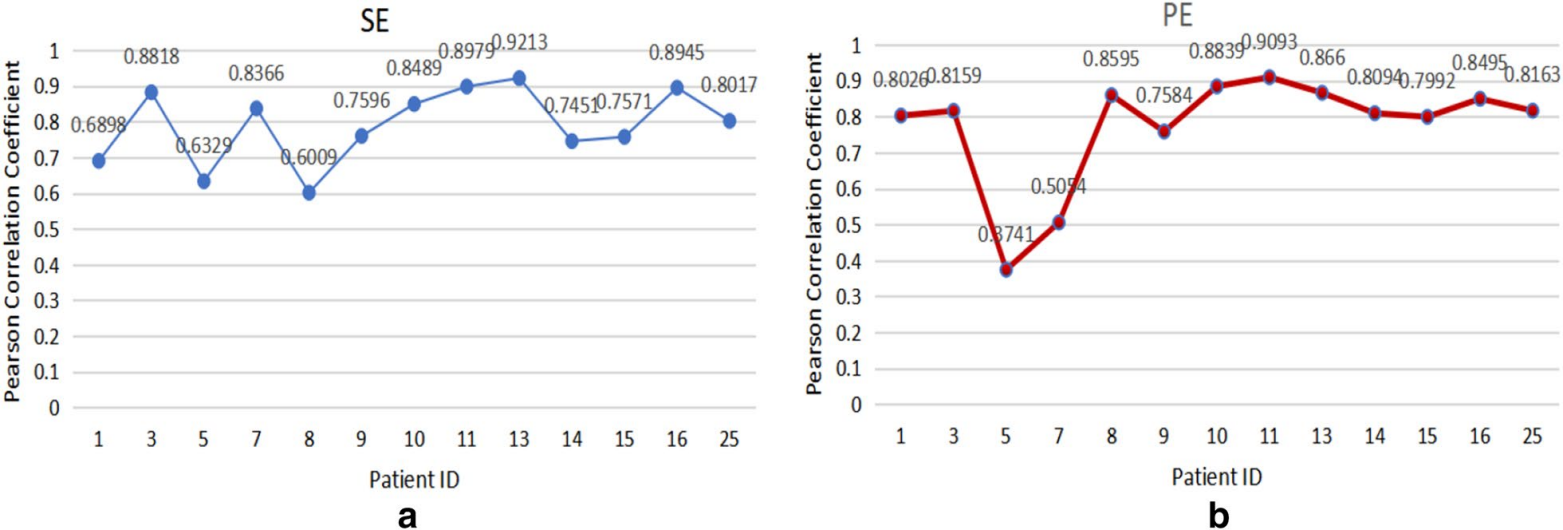
*r* values: **a** SE calculated from *β* and *βγ* frequency bands vs. BIS Index; **b** PE calculated from *γ* and *β1* frequency bands vs. BIS index

The permutation entropy (PE) feature extraction method is widely applied for the assessment of the depth of anaesthesia. Many researchers have proved that PE value is one reliable parameter for accurate DoA assessment [31–34]. Therefore, to evaluate the performance of SE parameters, PE values are also calculated with the same process as the SE values in the following experiments, and the comparison results are presented in Figs. 2, 3, 4,  5.

### Features selection

There are two frontal channels (Ch1 and Ch2) from which EEG signals are recorded and collected in the datasets. To reduce the features’ dimension, channel selection is an essential step of data analysis. SE and PE values are calculated from EEG signals of channel 2 (Ch2) and the sum of EEG signals of channel 1 and channel 2 (Ch1 + Ch2) to select one of them so that the experimental design can be simplified and more efficient. As shown in Fig. 2, the *R*^*2*^ values of SE and PE from Ch2 and Ch1 + Ch2 (the reference is the BIS index) are very close. Therefore, it is not necessary to use the EEG signals from both Ch2 and Ch1 + Ch2. Thus the EEG signals from Ch2 are analysed in this research.

The EEG signals from Ch2 are firstly decomposed into basic frequency bands (*α, β, γ, δ* and *θ*) and small frequency bands. As a result, ten sets of frequency bands from each episode of EEG signals are obtained. The SE and PE values are calculated based on both the amplitude and power of each basic frequency band. The scatter plot graphs for the SE and PE values and the BIS value show that SE and PE are roughly linearly correlated with the BIS index, as shown in Fig. 3.

The average *R*^*2*^ values of the SE and PE in each frequency band of the EEG signals from 13 patients (randomly chosen) are shown in Fig. 4.

The highest *R*^*2*^ calculated from the SE of *βγ* is 0.8459, whereas the highest *R*^*2*^ for the PE is 0.7927 (Fig. 4). Therefore, the most suitable feature parameters from different frequency bands for the DoA assessment using time-domain methods in this study is:

- The SE values, which are calculated from the amplitude of the *βγ* frequency band

To increase the robustness of the new DoA index, the SE values with the second highest *R*^*2*^, calculated from the amplitude of the *β* frequency band, are also applied for the index design.

**Fig. 6:**
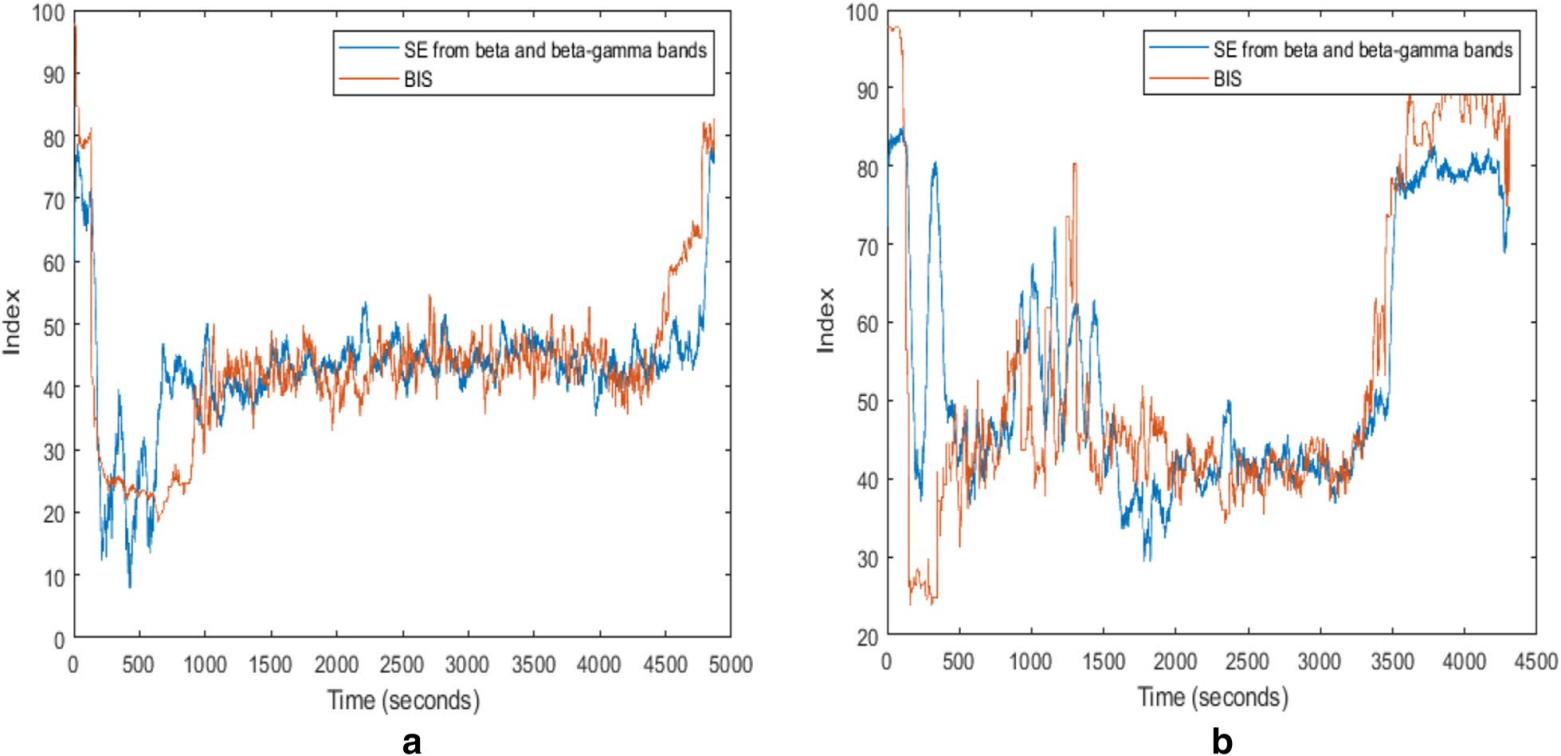
The index from the linear regression equation of the SE calculated from *β* and *βγ* frequency bands vs. the BIS Index. **a** Patient ID 9; **b** Patient ID 25

The proposed new DoA index based on the time characteristics is designed using these two parameters. The simple linear regression analysis exhibits that SE values calculated from *β* and *βγ* frequency bands are significantly linearly related to the BIS index. The highest value of *r* from the linear regression is 0.9213 (Patient ID 13) (Fig. 5(a)). The PE values calculated from *γ* and *β1* frequency bands are also strongly linearly related to the BIS index in some cases, but not consistently highly related to the BIS index across the patients (Patient ID 5 and ID 7 in Fig. 5b). Correlation analysis provides information on the strength and direction of the linear relationship between two variables, while a simple linear regression analysis estimates parameters in a linear equation that can be used to predict the values of one variable based on the other. The trends of the index from the linear regression equation of the SE calculated from *β* and *βγ* frequency bands show a great similarity with the BIS index (Fig. 6).

### Models based on regression analysis

**Fig. 7:**
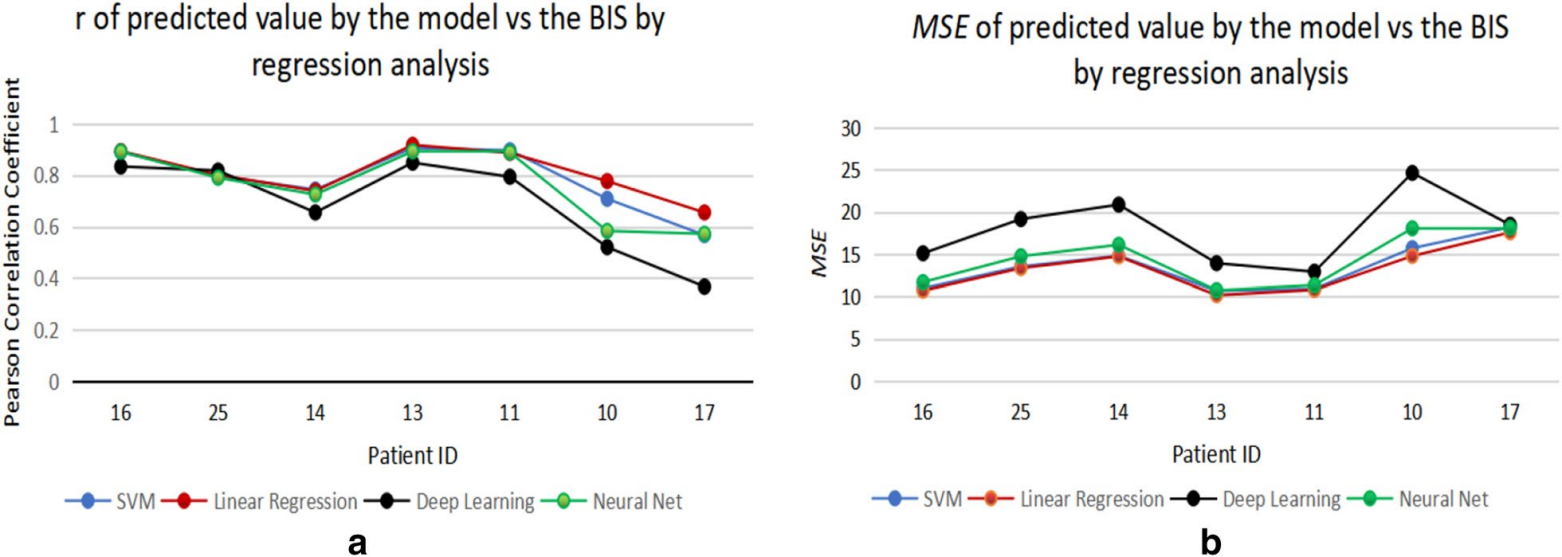
**a** *r* of predicted value by the model vs the BIS by regression analysis. **b** *RMSE* of predicted value by the model vs the BIS by regression analysis

Four methods of a linear regression, an SVM, a deep learning algorithm and a neural network were employed to select the most suitable model for the DoA measurement, and the linear regression analysis was proved to be the best analytical model for the DoA assessment based on the Pearson correlation coefficient (*r*), the root mean squared error (*RMSE*) and execution time in this study. To determine the method of analysis, ten sets of subjects of the selected parameters (the SE calculated from *β* and *βγ*) are trained by four candidates of regression models, and randomly chosen seven sets of those were tested (Patient IDs: 10, 11, 13, 14, 16, 17 and 25). The predicted values by the models are evaluated by comparing them with the BIS index. *r* and *RMSE* are used to examine the correlation of the predicted value and the BIS index. The results are shown in Fig. 7.

From Fig. 7, *r* values from linear regression analysis are higher (more correlated with the BIS) than those from other methods, SVM (kernel type: polynomial, Kernel degree: 2.0, kernel cache: 200, max iterations: 100,000), deep learning (activation: rectifier, hidden layer sizes: 50, epochs: 10), and neural network (training cycles: 200, learning rate: 0.01, momentum: 0.9). The highest *r* from linear regression analysis is 0.914 (Fig. 7a). In addition, the *RMSE* from the linear regression analysis is lower than any other analytical methods in this study. The lowest *RMSE* from the linear regression of these samples is 10.16 (Fig. 7b). The execution time is also an essential factor in selecting analytical methods because the execution time for analysis is crucial for real-time measurements of the DoA. The average execution time for each regression analysis is measured and shown in Table 2. The linear regression analysis records the shortest execution duration (0.5 s), and the SVM takes the longest time for the analysis (186 s).

**Table 2:** The average execution time of regression analysis (simulated by RapidMiner version 9.4)

	SVM	Linear regression	Deep learning	Neural net
Average execution time	186 s	0.5 s	4 s	6 s

### The new DoA design and evaluation by linear regression

The selected parameters (SE calculated from *β* and *βγ*) of the EEG data from ten subjects (Patient IDs: 1, 2, 4, 5, 6, 8, 15, 20, 22 and 23. Each record contains 18,448 s of EEG data, having 2,361,344 data points) are used to obtain the coefficients for a new DoA index, which employs the linear regression model. The new index is evaluated by comparing it with the BIS index. *r* values are used to examine the correlation between the new index and the BIS index. The new DoA index is proposed as follows:

9 $${\text{New DoA Index }} = 0.{2}0{9 }*{\text{ SE}}\_\beta + \, 0.{51}0 \, *{\text{ SE}}\_\beta \gamma ,$$

where SE_ *β* is the SE values calculated from *β* frequency band (13–30 Hz) and SE_ *βγ* is the SE values calculated from *βγ* frequency band (21.5–38.5 Hz).

**Fig. 8:**
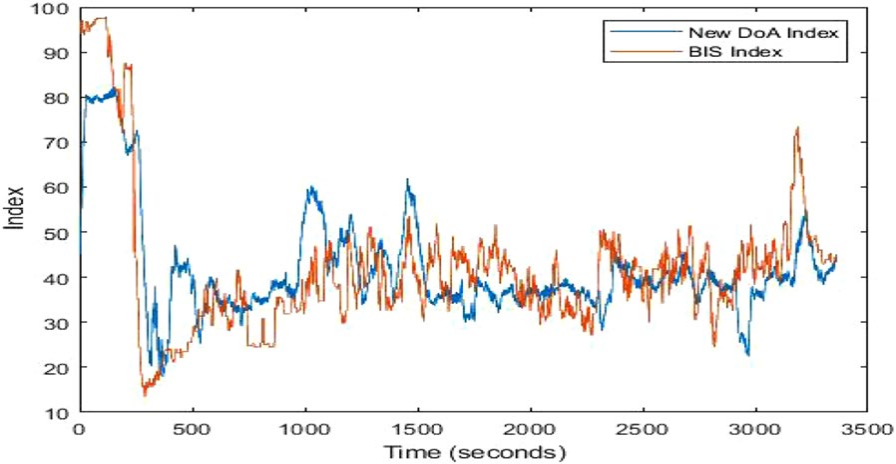
The new DoA index (Patient ID 16, *r* = 0.894) and the BIS index

The new DoA index (Patient ID 16, *r* = 0.893) and the BIS index are shown in Fig. 8. The trend of the new DoA index line shows a close similarity with the BIS index, having fewer fluctuations.

**Fig. 9:**
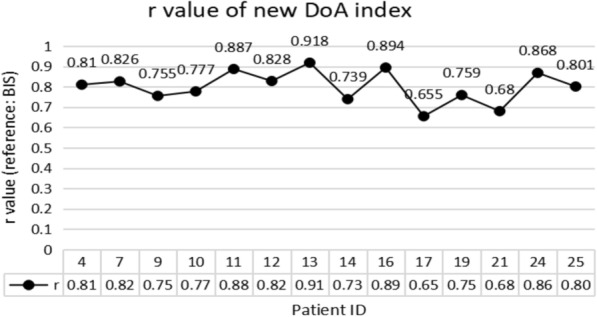
The performances of the new DoA index for randomly selected 14 patients (*r* values)

The performances of the new DoA index for randomly selected 14 patients (23,288 s which contain 2,980,864 data) are evaluated. *r* values for the 14 cases are shown in Fig. 9.

The average *r* values for the 14 patients is 0.8079, and the highest score is 0.914. The lowest *RMSE* is 8.62. The high *r* values show a very close correlation between the proposed index and the BIS index. However, the performance of the two cases (Patient IDs 17 and 21) was not good enough (*r* values are 0.65 and 0.68). The poor performance can be explained by the poor signal quality of the EEG from the two subjects (Patient ID 17 and 21).

### Patient’s state in the case of poor signal quality

**Fig. 10:**
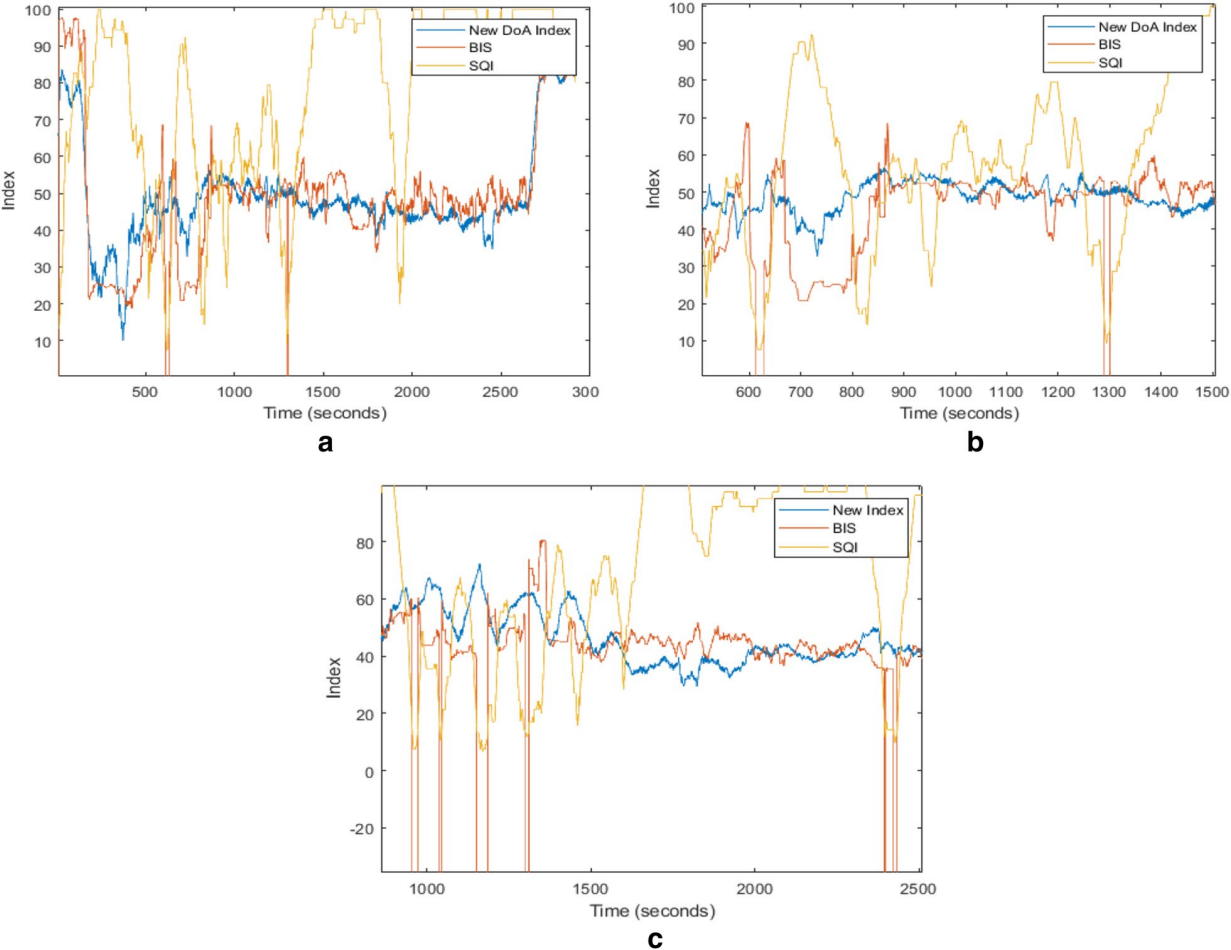
Comparisons of the new index and the BIS index with SQI. **a**. Patient ID: 19 range: 0–3000 s; **b** Patient ID: 19, range: 500–1500 s; **c** Patient ID: 25, range: 800–2300 s

The signal quality indicator (SQI) is an index for signal quality which is calculated based on impedance data, artefacts, and other variables. The BIS index is not capable of calculating the valid values on the screen when SQI is lower than 15. In these cases, the value − 3276.8 was labelled as a notice “excessive artefact detected in signal” [35]. The index of the proposed method in poor signal quality cases (according to SQI) is also evaluated. The new index produces the DoA values when SQI is lower than 15, where the BIS index could not calculate it. In Fig. 10, for Patient ID 19, the BIS index is −3276.8 from 611 to 629 s and from 1294 to 1301 s. For Patient ID 25, the BIS index is −3276.8 from 956 to 971 s, from 1040 to 1045 s, from 1153 to 1185 s, from 1299 to 1310 s and from 2396 to 2433 s. However, the new index shows the measured DoA value clearly during those periods. Along with the anaesthetists' records, there was no alteration in the patient’s anaesthetic states during this period. Consequently, the new index is more consistent to show the changes from one anaesthetic state to another state.

### Time delay from deep anaesthetic state to moderate anaesthetic state

**Fig. 11:**
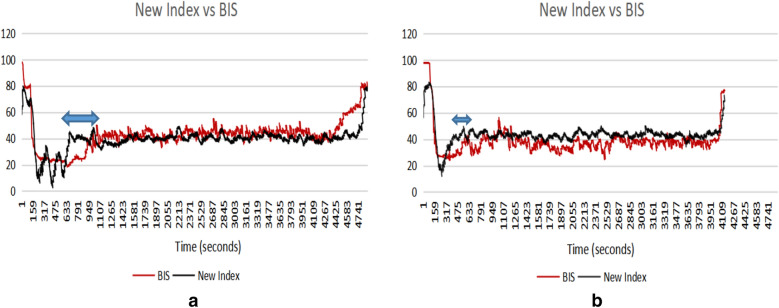
The comparison of the new index and the BIS index. **a** Patient ID 9; **b** Patient ID 12. The blue markers show the earlier reaction by the new index

The new proposed index shows a high correlation with the BIS throughout the states of consciousness, light anaesthesia and deep anaesthesia. It shows an earlier reaction than the BIS index when the patient from deep anaesthesia to moderate anaesthesia. This type of earlier reaction exists in all the cases of the 14 subjects. The new index for Patient IDs 9 and 12 are selected as examples to show the time response differences with the BIS index graphically in Fig. 10. The index value 35 is assumed to be the point at which the anaesthetic states transfers from deep anaesthesia to moderate anaesthesia. Comparing with the new index, BIS values show an average 158 s time delay of anaesthetic states changes for 14 patients, as shown in Fig. 11 and Table 3. The time differences for 14 patients are provided in Table 3.

**Table 3:** The time response comparison between the new index and the BIS (in seconds)

Patient ID	4	7	9	10	11	12	13	14	16	17	19	21	24	25
Time difference	200	75	258	231	41	116	331	100	6	157	288	266	132	14

## Discussion

The extracted features reduced the number of data points needed to describe a huge set of data effectively, as well as to minimize the loss of essential information embedded in the signals. The features extraction based on the SE in this study successfully leads to develop a reliable DoA algorithm for accurate DoA assessment. The denoised EEG signals were, firstly, divided into ten sub-frequency bands (*α, β1, β2, β3, β4, β, βγ, γ, δ,* and *θ*), and then the basic complexity measure was done by using SE and PE. The SE from *βγ* frequency band and the SE from the *β* frequency band yield the highest *R*^*2*^ value (0.8458 and 0.7312, respectively) with the BIS in this study. The FFT enabled frequency bands decomposition from the EEG signals, and the SE values were obtained from the ten sub-frequency bands. This study proves that the results of the experiment by Xu et al. [21], which showed that the SE was sensitive to the states of rats’ light and deep sleeps. Some studies [31–34] proposed that PE is a promising feature for discriminating different levels of consciousness during anaesthesia. The PE showed a high correlation (the highest *R*^2^ = 0.793) with the BIS index, but the SE presented an improved correlation (the highest *R*^2^ = 0.846) than the PE in this study. Along with other studies related to the analyses of the human brain activity [19, 36], the SE was proved to be a promising algorithm to monitor the stages of human anaesthesia during surgery.

## Conclusions

The new DoA index was developed based on two SE values (from *β* and *βγ*) for monitoring the DoA. It was evaluated by comparing it with the BIS index and the clinical observations by an attending anaesthetist. The highest *r* value is 0.918, and its average value is 0.80. The lowest *RMSE* is 8.62. The high *r* values indicate the proposed DoA index highly correlates with the BIS index. Furthermore, the proposed index responds to the changes in EEG signals better than the BIS index when a patient from deep anaesthesia to moderate anaesthesia. The new index shows the earlier time response (average 158 s for 14 patients) of states change than the BIS index.

The proposed DoA index demonstrates its consistency in the case of poor signal quality (SQI < 15) as well, while the BIS Index exhibits inflexibility with cases of poor signal quality (SQ). Some cases of our simulations exhibit poor correlations with the BIS, which may be due to the BIS index is inflexible with cases of poor signal quality. Four machine learning methods of an SVM, a neural network, a deep learning and a linear regression were employed to evaluate the accuracy of their DoA assessment. The linear regression outperformed the other three methods not only in accuracy (average *r* = 0.80), but also with a shorter execution time. The linear regression took only 0.5 s to simulate more than 35,000 data (run by RapidMiner version 9.4). The new DoA index by linear regression provides potential benefits as good guidance in real-time DoA assessments.

## Data Availability

The data and materials used in this study are available at the University of Southern Queensland under the research data management policy.

## References

[1] Diykh M, Li Y, Wen P, Li T (2018). Complex networks approach for depth of anesthesia assessment. Measurement.

[2] Nguyen-Ky T, Wen P, Li Y (2009). Theoretical basis for identification of different anesthetic states based on routinely recorded EEG during operation. Computers in Biology and Medicine.

[3] Ahmadi B, Negahbani E, Amirfattahi R, Zaghari B, Mansouri M (2008) Extraction of BIS™ index sub-parameters in different anesthetic and sedative levels. In: 9th International Conference on Signal Processing. IEEE, 2008, pp 2665–2668

[4] Iselin-Chaves IA, Willems SJ, Jermann FC, Forster A, Adam SR, Van der Linden M (2005). Investigation of implicit memory during isoflurane anesthesia for elective surgery using the process dissociation procedure. Anesthesiol J Am Soc Anesthesiologists.

[5] Musizza B, Ribaric S (2010). Monitoring the depth of anaesthesia. Sensors.

[6] Zanin M, Zunino L, Rosso OA, Papo D (2012). Permutation entropy and its main biomedical and econophysics applications: a review. Entropy.

[7] Siuly NA, Li Y, Wen P (2013). Identification of motor imagery tasks through CC-LR algorithm in brain computer interface. Int J Bioinform Res Appl.

[8] Suk H-I, Lee S-W (2013). A novel bayesian framework for discriminative feature extraction in brain-computer interfaces. IEEE Trans Pattern Anal Mach Intelligence.

[9] Zhang Y, Wang Y, Jin J, Wang X (2017). Sparse Bayesian learning for obtaining sparsity of EEG frequency bands based feature vectors in motor imagery classification. Int J Neural Syst.

[10] Faust O, Acharya UR, Adeli H, Adeli A (2015) Wavelet-based EEG processing for computer-aided seizure detection and epilepsy diagnosis. Seizure 26:56-64. 10.1016/j.seizure.2015.01.012 25799903

[11] Lee S-H, Lim JS, Kim J-K, Yang J, Lee Y (2014). Classification of normal and epileptic seizure EEG signals using wavelet transform, phase-space reconstruction, and Euclidean distance. Comp Methods Programs Biomed.

[12] Murugappan M. and Murugappan S. (2013) Human emotion recognition through short time Electroencephalogram (EEG) signals using Fast Fourier Transform (FFT). In: 2013 IEEE 9th International Colloquium on Signal Processing and its Applications. IEEE, Kuala Lumpur, pp 289–294. http://ieeexplore.ieee.org/document/6530058/.

[13] Kusumandari D, Suhendra M, Amri M, Simbolon A, Rizqyawan M, Wardono P, Fauzan A, Turnip A (2018) Comparison of EEG sleep characteristic with music and aromatherapy stimuli. Journal of Physics: Conference Series. IOP Publishing, p 012050

[14] Liang Z, Wang Y, Sun X, Li D, Voss LJ, Sleigh JW, Hagihira S, Li X (2015). EEG entropy measures in anesthesia. Front Comput Neurosci.

[15] Nguyen-KY T, Peng W, Yan L (2010). An improved detrended moving-average method for monitoring the depth of anesthesia. IEEE Trans Biomed Eng.

[16] Kortelainen J, Väyrynen E, Seppänen T (2011). Isomap approach to EEG-based assessment of neurophysiological changes during anesthesia. IEEE Trans Neural Syst Rehabil Eng.

[17] Wu W, Nagarajan S, Chen Z (2015). Bayesian Machine Learning: EEG/MEG signal processing measurements. IEEE Signal Process Mag.

[18] Burke DP, Kelly SP, deChazal P, Reilly RB, Finucane C (2005) A parametric feature extraction and classification strategy for brain–computer interfacing. IEEE Trans Neural Syst Rehabil Eng 13(1):12–17. http://ieeexplore.ieee.org/document/1406016/10.1109/TNSRE.2004.841881 15813401

[19] Zhang R, Xu P, Chen R, Li F, Guo L, Li P, Zhang T, Yao D (2015). Predicting inter-session performance of SMR-based brain-computer interface using the spectral entropy of resting-state EEG. Brain Topogr.

[20] Das AB, Bhuiyan MIH (2016). Discrimination and classification of focal and non-focal EEG signals using entropy-based features in the EMD-DWT domain. Biomed Signal Process Control.

[21] Xu J, Zheng C, Liu X, Pei X, Jing G (2006) Detecting brain activity variation of rat during anaesthesia by spectral entropy. In: 2005 IEEE engineering in medicine and biology 27th annual conference. IEEE, pp 6985–6988 10.1109/IEMBS.2005.1616113 17281882

[22] Ulrych TJ (1972). Maximum entropy power spectrum of truncated sinusoids. J Geophys Res.

[23] Lewis-Beck MS, Skalaban A (1990). The R-squared: some straight talk. Polit Anal.

[24] Yildirim P, Birant KU, Radevski V, Kut A, Birant D (2018) Comparative analysis of ensemble learning methods for signal classification. 2018 26th Signal Processing and Communications Applications Conference (SIU). IEEE, Izmir, pp. 1–4. https://ieeexplore.ieee.org/document/8404601/

[25] Liang Z, Huang C, Li Y, Hight DF, Voss LJ, Sleigh JW, Li X, Bai Y (2018). Emergence EEG pattern classification in sevoflurane anesthesia. Physiol Meas.

[26] Kumar A, Anand S (2006). A depth of anaesthesia index from linear regression of eeg parameters. J Clin Monitor Comput.

[27] Liu Q, Ma L, Fan S-Z, Abbod MF, Shieh J-S (2018). Sample entropy analysis for the estimating depth of anaesthesia through human EEG signal at different levels of unconsciousness during surgeries. PeerJ.

[28] Pearson K (1930). The life, letters and labors of francis galton.

[29] Marmolin H (1986). Subjective MSE measures. IEEE Trans Syst Man Cybern.

[30] Kertai MD, Whitlock EL, Avidan MS (2012). Brain monitoring with electroencephalography and the electroencephalogram-derived bispectral index during cardiac surgery. Anesthesia Analgesia.

[31] Jordan D, Stockmanns G, Kochs EF, Pilge S, Schneider G (2008). Electroencephalographic order pattern analysis for the separation of consciousness and unconsciousness: an analysis of approximate entropy, permutation entropy, recurrence rate, and phase coupling of order recurrence plots. Anesthesiology.

[32] Olofsen E, Sleigh J, Dahan A (2008) Permutation entropy of the electroencephalogram: a measure of anaesthetic drug effect. British J Anaesth 101:810–821 10.1093/bja/aen290 18852113

[33] Silva A, Campos S, Monteiro J, Venâncio C, Costa B, de Pinho PG, Antunes L (2011). Performance of anaesthetic depth indexes in rabbits under propofol anaesthesia: prediction probabilities and concentration-effect relations. Anaesthesiology.

[34] Morabito FC, Labate D, La Foresta F, Bramanti A, Morabito G, Palamara I (2012) Multivariate multi-scale permutation entropy for complexity analysis of Alzheimer’s disease EEG. Entropy 14(7):1186-1202.

[35] Nguyen-Ky T, Wen P, Li Y (2013). Consciousness and depth of anesthesia assessment based on bayesian analysis of EEG signals. IEEE Trans Biomed Eng.

[36] Raghu S, Sriraam N (2017). Optimal configuration of multilayer perceptron neural network classifier for recognition of intracranial epileptic seizures. Expert Syst Appl.

